# Anti-Obesity Activity of *Sanghuangporus vaninii* by Inhibiting Inflammation in Mice Fed a High-Fat Diet

**DOI:** 10.3390/nu16132159

**Published:** 2024-07-06

**Authors:** Jie Hao, Xinghui Jin, Zhige Li, Yanfeng Zhu, Lu Wang, Xue Jiang, Di Wang, Liangliang Qi, Dongxu Jia, Bo Gao

**Affiliations:** 1 School of Life Sciences, Jilin University, Changchun 130012, China; haojie21@mails.jlu.edu.cn (J.H.); jinxh21@mails.jlu.edu.cn (X.J.); lizg21@mails.jlu.edu.cn (Z.L.); zhuyf21@mails.jlu.edu.cn (Y.Z.); wanglu1319@mails.jlu.edu.cn (L.W.); jluwangdi@jlu.edu.cn (D.W.); 2 College of Life Science and Technology, Changchun University of Science and Technology, Changchun 130022, China; jiangxue@cust.edu.cn; 3 Engineering Research Center of Chinese Ministry of Education for Edible and Medicinal Fungi, Jilin Agricultural University, Changchun 130118, China; 4 Microbiology Research Institute, Guangxi Academy of Agricultural Sciences, Nanning 530007, China; gx_macrofungi@gxaas.net

**Keywords:** *Sanghuangpours vaninii*, obesity, lipids, inflammation, TLR4/NF-κB

## Abstract

Obesity is an unhealthy condition associated with various diseases characterized by excess fat accumulation. However, in China, the prevalence of obesity is 14.1%, and it remains challenging to achieve weight loss or resolve this issue through clinical interventions. *Sanghuangpours vaninii* (SPV) is a nutritional fungus with multiple pharmacological activities and serves as an ideal dietary intervention for combating obesity. In this study, a long-term high-fat diet (HFD) was administered to induce obesity in mice. Different doses of SPV and the positive drug simvastatin (SV) were administered to mice to explore their potential anti-obesity effects. SPV regulated weight, serum lipids, and adipocyte size while inhibiting inflammation and hepatic steatosis. Compared with the vehicle-treated HFD-fed mice, the lowest decreases in total cholesterol (TC), triglycerides (TG), and low-density lipoprotein cholesterol (LDL-C) were 9.72%, 9.29%, and 12.29%, respectively, and the lowest increase in high-density lipoprotein cholesterol (HDL-C) was 5.88% after treatment with different doses of SPV. With SPV treatment, the analysis of gut microbiota and serum lipids revealed a significant association between lipids and inflammation-related factors, specifically sphingomyelin. Moreover, Western blotting results showed that SPV regulated the toll-like receptor (TLR4)/nuclear factor kappa B (NF-κB) signaling pathway in HFD-diet mice, which is related to inflammation and lipid metabolism. This research presents empirical proof of the impact of SPV therapy on obesity conditions.

## 1. Introduction

Obesity is a multifactorial disease [1] that is linked to a higher prevalence of numerous severe health issues, such as type 2 diabetes mellitus, dyslipidemia, and nonalcoholic fatty liver disease [2,3]. A cross-sectional study in 2023 revealed that the overweight rate in China reached 34.8%, and the obesity rate reached 14.1% (based on Chinese BMI classification) among 15.8 million adults [4]. The prevalence of obesity has been increasing at an alarming speed, which places a huge burden on China’s healthcare system [5].

Chronic tissue inflammation is a key feature of obesity [6]. Tumor necrosis factor-alpha (TNF-α) is a cytokine with multiple functions, and its elevation is often associated with inflammatory diseases. It has been found to be increased in adipose tissue from obesity [7]. TNF-α can activate intracellular signaling molecules, such as inhibitory kappa B kinase (IKK) β, which impairs insulin resistance and leads to nuclear factor kappa B (NF-κB) nuclear translocation, further driving an increase in inflammatory mediators [8]. Inflammasomes are usually involved in the activation of inflammation, and the nucleotide-binding oligomerization domain leucine-rich repeat and pyrin domain containing 3 (NLRP3) inflammasome induce the release of caspase-1-mediated inflammatory cytokines, subsequently increasing the level of interleukin (IL)-1β [9]. IL-1β is closely related to insulin secretion and glucose metabolism [10]. Furthermore, obesity is also associated with changes in gut microbiota and dysregulation of lipid metabolism [11,12]. The gut microbiota not only regulates fat storage but also affects inflammation, insulin, and glucose metabolism [13]. For example, short-chain fatty acids (SCFAs), which are metabolites of gut microbes, can inhibit fat production, reduce lipid accumulation, and increase thermogenesis to decrease adiposity [14]. SCFAs are also capable of suppressing the activation of NLRP3 inflammasomes and the secretion of inflammatory cytokines in cells [15]. Therefore, inhibiting NLRP3 overactivation and improving gut microbiota can regulate lipid metabolism and alleviate symptoms of obesity.

Currently, the main methods for alleviating symptoms of obesity include lifestyle intervention, the consumption of anti-obesity drugs, and bariatric surgery [16]. While short-term weight loss may be achieved by restricting food intake, subsequent compensatory physiological adaptations often lead to weight regain [17]. Orlistat is a drug that can be used for long-term treatment of obesity by reducing gastrointestinal absorption of meals in order to achieve weight reduction. However, it is frequently associated with gastrointestinal and liver toxicity [18]. Although the safety of bariatric surgery has greatly improved compared to the past, there still exists a possibility of postoperative infection and complications [19]. Therefore, there is an urgent need for a new treatment strategy for obesity. Macrofungi have gained widespread attention due to their diverse pharmacological activities [20]. *Sanghuangporus vaninii* (SPV) is a fungus that lives in wood and has been extensively utilized in traditional Chinese medicine [21]. Studies have shown that the polysaccharides of SPV can alleviate hyperglycemia and hyperlipidemia by regulating gut microbiota in type 2 diabetic mice [22]. Furthermore, SPV polysaccharides can act as anti-tumor agents against colorectal cancer by enhancing the function of Th1 cells and reducing the inhibitory effect of Th2 cells [23]. The anti-inflammatory properties of SPV’s fruit bodies can help reduce hyperuricemia and gouty arthritis [24]. Additionally, extracts from SPV have demonstrated anti-cancer activity in human cervical cancer SiHa cells [25]. However, the effects of SPV fruiting bodies on diet-induced obese mice have not been systematically studied.

In this study, we systemically demonstrated the protective role of SPV in in mice fed a high-fat diet (HFD), specifically modulating the lipid metabolism process particularly associated with ceramides (Cer), and protecting against inflammation via the toll-like receptor (TLR4)/NF-κB signaling pathway mostly based on gut microbiota, plasma lipidome analysis, and biochemical detection.

## 2. Materials and Methods

### 2.1. SPV Preparation

Fruiting bodies of SPV were collected from “Sanghuang Town” in Bajiazi Town, Jilin, China. The fruiting bodies were washed, dried, crushed into coarse powders, ground into ultrafine powders, and stored under dry conditions at room temperature before SPV administration.

### 2.2. Animal Experimental Protocols

**Figure 1:**
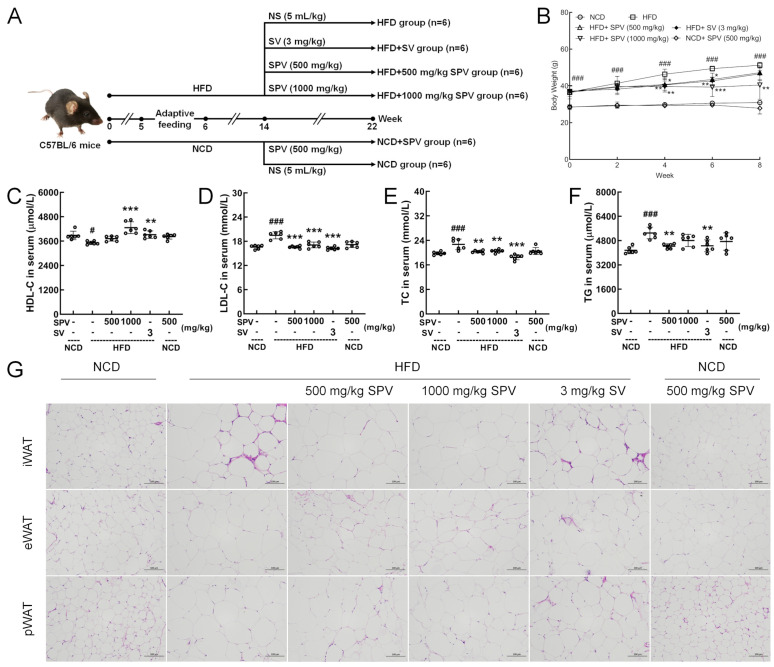
SPV alleviated HFD-induced obesity and hyperlipidemia. (**A**) Establishment of animal models and agent administration management. (**B**) SPV suppressed body weight gain in HFD-fed mice (*n* = 6). SPV administration led to a rise in the serum level of (**C**) HDL-C and a reduction in the serum levels of lipid markers (**D**) LDL-C, (**E**) TC, and (**F**) TG in the HFD-fed mice (*n* = 6). (**G**) H&E staining of iWAT, eWAT, and pWAT (200×, scale bar: 100 μm). The data are presented as the mean ± SD. ^#^ *p* < 0.05, ^###^ *p* < 0.001 versus the vehicle-treated NCD-fed mice; * *p* < 0.05, ** *p* < 0.01, *** *p* < 0.001 versus the vehicle-treated HFD-fed mice.

Thirty-six male C57BL/6JGpt mice, provided by GemPharmatech Co., Ltd. (Nanjing, China; SCXK [SU] 2018-0008), were accommodated in stable conditions with a maintained temperature of 23 ± 1 °C and humidity ranging from 40% to 60%. They were kept on a 12-h light/dark cycle and had ad libitum access to food. The mice were divided into two groups in a random manner; for eight weeks, 24 mice received the HFD feed (D12492), while 12 mice received the normal chow diet (NCD; D12450B). After eight weeks, the 24 mice fed with a high-fat diet were divided into four groups at random (*n* = 6). They were orally administered either 500 mg/kg SPV (HFD + 500 mg/kg SPV group), or 1000 mg/kg SPV (HFD + 1000 mg/kg SPV group), or 3 mg/kg simvastatin (SV) (Chengdu Hengrui Pharmaceutical Co., Ltd., Chengdu, China) (HFD + SV group), or 5 mL/kg normal saline (HFD group). Meanwhile, the 12 mice fed with the NCD were then divided into two groups at random (*n* = 6). They received either an oral dose of 500 mg/kg SPV (NCD + SPV group) or 5 mL/kg normal saline (NCD group) for an additional 8 weeks (Figure 1A), and a single animal is the experimental unit. After the last administration in the 8-week administration period, all mice were fasted for 8 h and provided with normal drinking water, then peripheral blood samples were collected from the tail vein before euthanizing the mice using CO_2_ inhalation. Following euthanasia, the heart, liver, spleen, kidney, epididymal white adipose tissue (eWAT), inguinal white adipose tissue (iWAT), and perirenal white adipose tissue (pWAT) were weighed and collected. All aforementioned samples were stored at −80 °C for further study.

### 2.3. Histopathological Analysis

#### 2.3.1. Hematoxylin and Eosin (H&E) Staining

The livers, hearts, spleens, kidneys, and three types of adipose tissue (eWAT, iWAT, and pWAT) were fixed with a tissue fixative solution (BL539A, Biosharp, Guangzhou, China). Then, the sections were treated with hematoxylin solution, bluing solution, and eosin solution before being dehydrated with ethanol, sealed with neutral gum, and observed under an inverted microscope.

#### 2.3.2. Oil Red O Staining

Liver sections fixed in tissue fixation solution were washed, and then immersed in the oil red dye solution. Subsequently, they were immersed in 60% isopropyl alcohol and pure water before undergoing hematoxylin staining. After being sealed with glycerin gelation, the sections were observed under a microscope.

### 2.4. Biochemical Detection

Frozen peripheral blood samples were incubated at room temperature for 30 min, and then centrifuged twice at 3500 rpm for 10 min to obtain serum samples. Levels of high-density lipoprotein cholesterol (HDL-C), low-density lipoprotein cholesterol (LDL-C), total cholesterol (TC), triglycerides (TG), leptin (LEP), and insulin (INS) in serum were detected using enzyme-linked immunosorbent assay (ELISA) kits. Per 30 mg samples of the liver were homogenized in 300 μL of saline. Supernatants were collected after centrifuging the homogenized liver samples twice at 3500 rpm. Levels of alanine aminotransferase (ALT), aspartate aminotransferase (AST), IL-18, TNF-α, IL-1β, IL-6, monocyte chemotactic protein-1 (MCP-1), free fatty acids (FFA), and Cer in the liver supernatants were detected with ELISA kits. The samples’ protein concentrations were measured with a bicinchoninic acid assay kit (23227; Thermo Fisher Scientific, Waltham, MA, USA) to provide support for quantification. The ELISA kits utilized are detailed in Table S1. The methodology is the same as our previous research [26].

### 2.5. Gut Microbiota Analysis

The contents of the cecum were gathered from three groups of mice (NCD group, HFD group, and HFD + 1000 mg/kg SPV group (HFD + SPV group)) as samples for the 16S rRNA analysis of gut microbiota. The samples underwent DNA isolation, determination of concentration and purity, confirmation of quality, and amplification of the 16S rRNA gene. The analysis was conducted on the platform provided by Shanghai Personal Biotechnology Co., Ltd. in Shanghai, China, as previously described [26].

The α-diversity was analyzed based on Chao1, Faith’s phylogenetic diversity (Faith’s PD), Good’s coverage, Simpson, and observed species. Meanwhile, Kruskal–Wallis rank-sum test and Dunn’s test were used for post hoc testing and significance testing, respectively. Principal coordinate analysis (PCoA) based on Bray-Curtis distance calculation can be employed for β-diversity analysis. The heat map with clustering trees of genus level of 20 bacteria in groups was generated to directly visualize differences between groups.

### 2.6. Plasma Lipidome Analysis

Serum samples from mice in the NCD group, HFD group, and HFD + 1000 mg/kg SPV group (HFD + SPV group) (*n* = 3) were prepared and analyzed using liquid chromatography–mass spectrometry (LC–MS) (LC, U300, Thermo USA) (MS, QE Focus Thermo USA) at Shanghai Personalbio Technology Co., Ltd. (Shanghai, China), as described in the earlier research [26]. Statistical analysis was performed using orthogonal partial least squares discriminant analysis (OPLS-DA) to identify differential lipidomes. Metabolite levels with statistically significant differences (*p* < 0.05) and variable importance in projection (VIP) values > 1.0 belonging to the lipidome were considered significantly differentially expressed for further analysis. The methodology is the same as our previous research [27].

### 2.7. Western Blotting

Livers were homogenized with radioimmunoprecipitation assay (RIPA) buffer (PC101, EpiZyme, Shanghai, China), which contained protease and phosphatase inhibitors (P002, New Cell & Molecular Biotech Co., Ltd., Jiangsu, China). Then, the homogenates were centrifuged to obtain supernatants as mentioned in Section 2.4 above. After denaturation and rationing samples with loading buffer and RIPA buffer, each sample containing 40 mg of protein was separated using 10% sodium dodecyl sulfate-polyacrylamide gel electrophoresis (SDS-PAGE) (PG112, Shanghai Epizyme Bio-medical Technology Co., Ltd., Shanghai, China) and transferred onto a membrane made of polyvinylidene fluoride (PVDF) (10600023, Cytiva, Marlborough, MA, USA). The membrane was blocked using a rapid closure solution (P30500, New Cell & Molecular Biotech Co., Ltd., Jiangsu, China), and then incubated overnight with the primary antibody solution followed by the secondary antibody solution for 4 h at 4 °C. Bands were visualized by ultra-high sensitivity enhanced chemiluminescence (ECL) kits (GK10008, GLPBIO, Montclair, NJ, USA) under an automated chemiluminescence image analysis system (Tanon 5200, Tanon Science & Technology Co., Ltd., Shanghai, China) as black fluorescence and quantified using ImageJ 1.54f (National Institutes of Health, Bethesda, MD, USA), and normalized to glyceraldehyde-3-phosphate de-hydrogenase (GAPDH). The antibodies used are listed in Table S2.

### 2.8. Statistical Analysis

All statistics are reported as the mean ± SD. Biochemical indices were compared among various groups using one-way analysis of variance (ANOVA), and then followed by Tukey’s test (except for gut microbiota analysis and plasma lipidome analysis). The tests were conducted using BONC DSS Statistics 25 (IBM, Armonk, NY, USA). *p* < 0.05 is regarded as a statistically significant difference.

## 3. Results

### 3.1. SPV Alleviated Obesity Induced by HFD

After the 8-week treatment period, the 1000 mg/kg SPV treatment significantly suppressed the increment in body weight in HFD-fed mice (*p* < 0.01, Figure 1B). However, treatment with 500 mg/kg SPV (*p* < 0.05, Figure 1B) and SV (*p* < 0.01, Figure 1B) only showed significant suppression of body weight increase for a duration of 6 weeks but not for 8 weeks (Figure 1B). The effect of regulating the body weights of HFD-fed mice was better with the 1000 mg/kg SPV treatment compared to either the treatment with 500 mg/kg SPV or SV. There was no significant effect observed in the body weights of NCD-fed mice following SPV treatment.

SPV treatment strongly up-regulated HDL-C (1000 mg/kg SPV, *p* < 0.001, Figure 1C) level and down-regulated LDL-C (*p* < 0.001, Figure 1D), TC (*p* < 0.01, Figure 1E) and TG (500 mg/kg SPV, *p* < 0.01, Figure 1F) levels in serum. No significant effects on HDL-C, LDL-C, TC, and TG levels in serum were observed among NCD-fed mice following SPV treatment.

The size of three types adipocytes decreased with both SPV treatment and SV treatment (Figure 1G and Figure S1), but no significant differences were found between SV treatment and two different concentrations of SPV treatment. No significant effects were observed on the size of three types of adipocytes in NCD-fed mice following SPV treatment.

### 3.2. SPV Alleviated Hepatic Steatosis and Inflammation in HFD-Fed Mice

**Figure 2:**
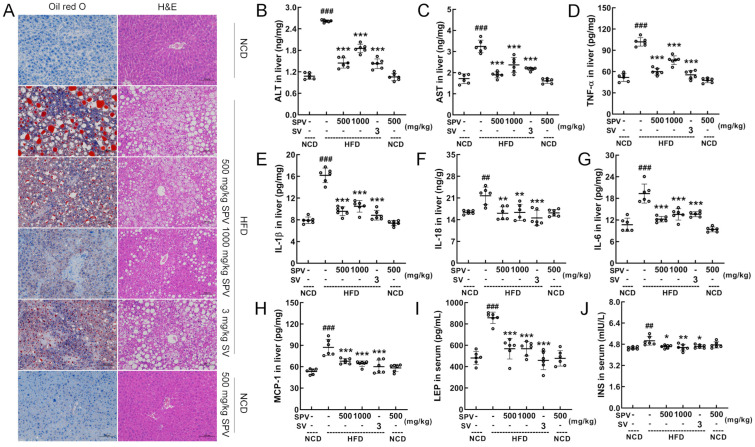
SPV alleviated hepatic steatosis and inflammation. (**A**) The liver was examined through histopathological methods using Oil Red O staining (200×; scale bar: 100 µm) and H&E staining (200×; scale bar: 100 µm). In the HFD-fed mice, SPV suppressed the liver levels of (**B**) ALT, (**C**) AST, (**D**) TNF-α, (**E**) IL-1β, (**F**) IL-18, (**G**) IL-6, and (**H**) MCP-1, and suppressed the serum levels of (**I**) LEP and (**J**) INS. The data are shown as the mean ± SD (*n* = 6). ^##^ *p* < 0.01, ^###^ *p* < 0.001 versus the vehicle-treated NCD-fed mice; * *p* < 0.05, ** *p* < 0.01, *** *p* < 0.001 versus the vehicle-treated HFD-fed mice.

Oil red O staining and H&E staining showed that SPV treatment reduced the lipid accumulation and lipid vacuoles induced by HFD feeding in the liver, with more significant effects observed in the 1000 mg/kg SPV treatment (Figure 2A). No difference or abnormalities were observed in the heart, spleen, and kidney (Figure S2). The levels of ALT (*p* < 0.001, Figure 2B) and AST (*p* < 0.001, Figure 2C), which are indicators of liver damage, were strongly down-regulated in the liver by SPV treatment. Compared with the vehicle-treated HFD-fed mice, levels of ALT in the livers of mice decreased by 44.77% and 29.17% after low and high doses of SPV administration, respectively. Similarly, levels of AST in the livers of mice decreased by 41.46% and 27.02% after low and high doses of SPV administration, respectively. Inflammation was significantly suppressed in SPV-treated HFD-fed mice. With the SPV treatment, levels of TNF-α (*p* < 0.001, Figure 2D), IL-1β (*p* < 0.001, Figure 2E), IL-18 (*p* < 0.01, Figure 2F), and IL-6 (*p* < 0.001, Figure 2G) were significantly down-regulated in the liver. Levels of TNF-α, IL-1β, IL-18, and IL-6 in the liver of HFD-fed mice decreased by 25.28%, 35.22%, 24.69%, and 29.92% at least, respectively, after administering different doses of SPV. Additionally, compared with the vehicle-treated HFD-fed mice, levels of MCP-1 (*p* < 0.001, Figure 2H) in the liver as well as LEP (*p* < 0.001, Figure 2I) and INS (*p* < 0.05, Figure 2J) in serum were down-regulated in SPV-treated mice. Specifically, levels of MCP-1 in the liver, LEP, and INS in the sera of mice decreased by 22.19%, 33.79%, and 8.76% at least, respectively.

### 3.3. SPV Strongly Influenced the Gut Microflora in HFD-Fed Mice

**Figure 3:**
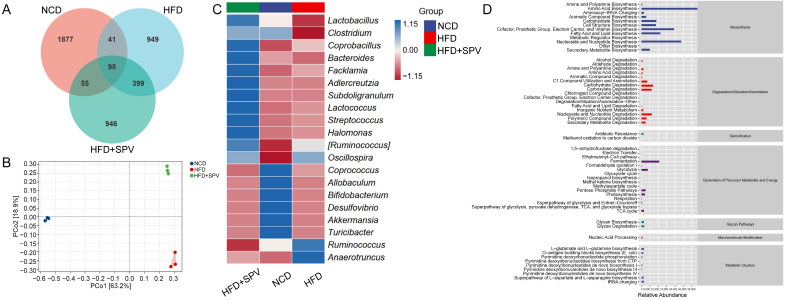
SPV regulated the gut microflora. (**A**) Venn diagram. (**B**) PCoA analysis. (**C**) Heatmap of the top 20 genera based on the average abundance values. (**D**) Graphical representation of the predicted abundances of secondary functional pathways derived from the MetaCyc database.

The OTU Venn diagram (Figure 3A) showed significant differences in the composition of gut microbiota among the three groups of mice (Unique Part: NCD: 43.03%, HFD: 21.76%, HFD + SPV: 21.69%, total: 86.47%). There was also a small overlap of 2.18% between the groups, indicating some similarity. Additionally, gut microbiota in SPV-treated mice were found to be more similar to HFD-fed mice (Figure S3). β-diversity analyzed with PCoA analysis revealed a long distance between the three groups, indicating substantial differences among them (Figure 3B). However, no significant differences were observed in α-diversity with SPV treatment (Figure S3A). Seven kinds of microbiota from various classes were down-regulated while 16 were up-regulated after SPV treatment in HFD-fed mice (Table S3). The heatmap visually displays the differences in relative abundance of 20 kinds of gut microbiota at the genus level among the three groups of mice (Figure 3C). Among these, SPV up-regulated the abundances of *Lactobacillus*, *Clostridium*, *Bacteroides*, *Adlercreutzia*, and *Allobaculum*, and down-regulated the abundances of *Anaerotruncus*, *Ruminococcus*, and *Oscillospira* in HFD-fed mice. Prediction of significantly different metabolic pathway based on MetaCyc showed 6 down-regulated pathways and 25 up-regulated pathways after SPV treatment in HFD-fed mice (*p* < 0.05, Table S4). Prediction of secondary functional pathways with relative abundance based on MetaCyc showed 58 pathways associated with gut microbiota, where biosynthesis is considered as the most important pathway type, including fatty acid and lipid biosynthesis (Figure 3D and Figure S4).

### 3.4. SPV Altered the Lipids Associated with Sphingomyelin (SM) in HFD-Fed Mice

**Figure 4:**
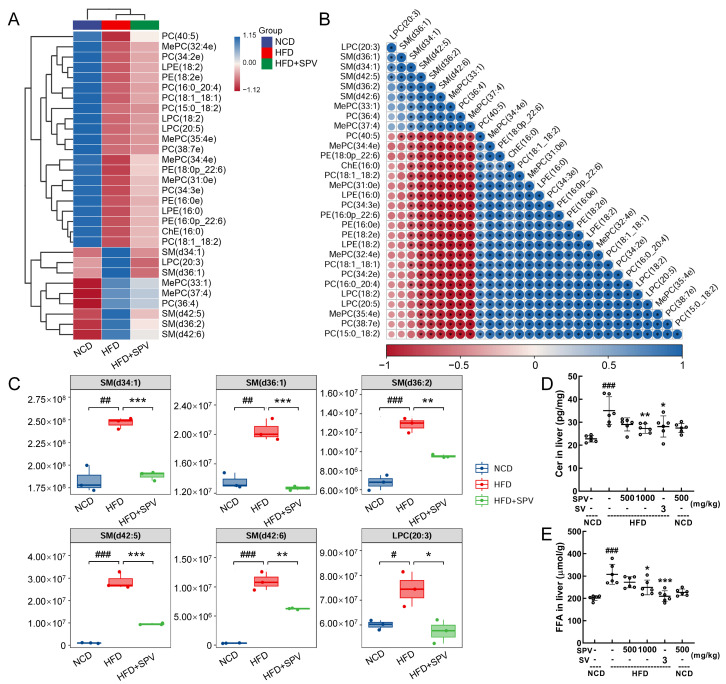
SPV regulated lipid metabolites in HFD-fed mice. (**A**) Heatmap of 30 significantly altered metabolites. (**B**) The associated heatmap of associated lipids. (**C**) Boxplots of 6 significantly altered metabolites in SPV-treated HFD-fed mice (*n* = 3). SPV treatment led to a decrease in the levels of (**D**) Cer and (**E**) FFA within the livers of HFD-fed mice (*n* = 6). ^#^ *p* < 0.05, ^##^ *p* < 0.01, ^###^ *p* < 0.001 versus the vehicle-treated NCD-fed mice; * *p* < 0.05, ** *p* < 0.01, *** *p* < 0.001 versus the vehicle-treated HFD-fed mice.

Thirty lipids were reversed by SPV treatment and are shown in the heatmap (Figure 4A and Figure S5) and correlation analysis map (Figure 4B and Figure S6) with their relative levels. In HFD-fed mice, SPV treatment significantly up-regulated 21 lipids and down-regulated 9 lipids (Table S5). Interestingly, among the down-regulated lipids, five belonged to SM (SM (d34:1), SM (d36:1), and SM (d42:5), *p* < 0.001; SM (d36:2) and SM (d42:6), *p* < 0.01 (Figure 4C) can be transformed into Cer by sphingomyelinases [26]. Cer (*p* < 0.01, Figure 4D) and FFA (*p* < 0.05, Figure 4E) were significantly down-regulated in the liver by 1000 mg/kg SPV treatment in HFD-fed mice.

### 3.5. SPV Regulated TLR4/NF-κB Signaling Pathway

**Figure 5:**
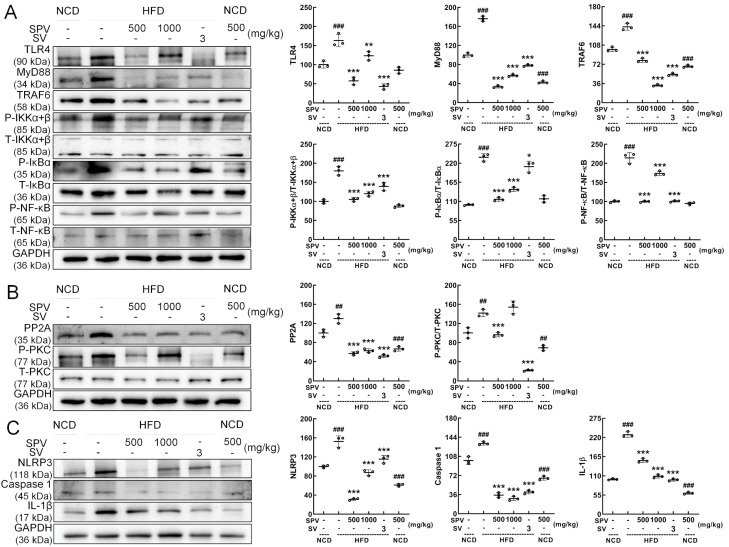
SPV regulated the TLR4/NF-κB signaling pathway in the livers of mice fed a high-fat diet. (**A**) SPV treatment resulted in reduced levels of TLR4, MyD88, and TRAF6 expression, and the phosphorylation levels of IKK α+β, IκBα, and NF-κB. (**B**) SPV treatment resulted in reduced expression level of PP2A and the phosphorylation level of PKC. (**C**) SPV treatment resulted in reduced expression levels of NLRP3, caspase 1, and IL-1β. Quantification data were normalized to glyceraldehyde-3-phosphate dehydrogenase (GAPDH) or the corresponding total protein concentration and expressed as the percentage of the vehicle-treated NCD-fed mice. The data are shown as the mean ± SD (*n* = 3). ^##^ *p* < 0.01 and ^###^ *p* < 0.001 versus the vehicle-treated NCD-fed mice; * *p* < 0.05, ** *p* < 0.01, *** *p* < 0.001 versus the vehicle-treated HFD-fed mice.

According to Western blot analysis, SPV significantly suppressed the expressions of TLR4 (*p* < 0.01), myeloid differentiation primary response protein 88 (MyD88) (*p* < 0.001), TNF receptor-associated factor 6 (TRAF6) (*p* < 0.001), p-IKKα+β (*p* < 0.001), p-NF-kappa-B inhibitor alpha (IκBα) (*p* < 0.001), and p-NF-κB (*p* < 0.001) in the livers of HFD-fed mice (Figure 5A). In order, compared with the vehicle-treated HFD-fed mice, the degrees of reduction are 24.50%, 67.65%, 44.18%, 33.30%, 39.02%, and 18.85% at least. SPV treatment also suppressed the expressions of protein phosphatase 2A (PP2A) (*p* < 0.001), and p-protein kinase C (PKC) (500 mg/kg SPV, *p* < 0.001) in the livers of HFD-fed mice (Figure 5B). Furthermore, SPV treatment also significantly suppressed the expressions of NLRP3 (*p* < 0.001), caspase-1 (*p* < 0.001), and IL-1β (*p* < 0.001) in the livers of HFD-fed mice (Figure 5C). In order, compared with the vehicle-treated HFD-fed mice, the degrees of reduction are 51.66%, 32.12%, 42.88%, 73.48%, and 32.62% at least.

## 4. Discussion

SPV fruiting bodies have various pharmacological activities, especially anti-tumor activity [28]. However, their anti-obesity effect has not yet been clarified. In this study, we systematically investigated the anti-obesity effect of SPV on HFD-fed mice and found that SPV achieved its anti-obesity effect and hypolipidemic activity by significantly reducing the body weight of high-fat mice and the serum levels of lipid markers LDL-C, TC, and TG, and notably increasing the level of HDL-C. This was also confirmed by the reduction in adipocytes in three types of adipose tissues. Additionally, SPV attenuated hepatic injury in HFD-fed mice with a reduced extent of hepatic steatosis and lipid infiltration. It also decreased levels of ALT and AST, which are hallmark factors for hepatic injury. Furthermore, SPV demonstrated potent anti-inflammatory action characterized by reduced levels of pro-inflammatory cytokines TNF-α, IL-1β, IL-18, and IL-6. In addition, levels of INS were significantly elevated in HFD-fed mice [29]. LEP is primarily produced in white adipose tissue, and chronic hyperinsulinemia leads to elevated LEP concentrations [30]. Human B cells stimulated with LEP exhibit elevated pro-inflammatory activities, such as increased expression of IL-6 and TNF-α [31], while MCP-1 is deeply expressed in inflammatory environments [32]. SPV significantly reduced the levels of MCP-1, LEP, and INS in HFD-fed mice. These findings suggest that SPV can modulate lipid metabolism by regulating anti-inflammatory functions. SPV is rich in a variety of active ingredients including polysaccharides and polyphenols, etc. The polysaccharides obtained from SPV have been reported to have hypolipidemic [22] and anti-cancer [23] activities, and the polyphenol extract may have anti-inflammatory activity [33]. It is hypothesized that SPV may act on some pathways through its active ingredients to achieve its anti-obesity efficacy.

The gut microbiota regulates energy metabolism in the host [34], and its dysfunction is heavily associated with obesity [35]. SPV treatment up-regulated the abundances of *Lactobacillus*, *Clostridium*, *Bacteroides*, *Adlercreutzia*, and *Allobaculum*, and down-regulated the abundances of *Anaerotruncus*, *Ruminococcus*, and *Oscillospira* in the gut microbiota. As a bacterium that promotes the production of SCFAs, *Lactobacillus* is recognized as a beneficial bacterium with anti-inflammatory, anti-insulin resistance, and anti-obesity properties. Its antimicrobial products in the gut can affect the gut microbiota by reducing intestinal permeability and maintaining intestinal homeostasis [36,37]. *Lactobacillus* activates the G protein-coupled receptor pathway while down-regulating the expression of NF-κB and TNF-α [37]. It also exerts a reparative effect on injury via inhibition of the PI3K/Akt pathway and additional inflammatory cytokines such as IL-1β, IL-6, IL-18, and MCP-1 [37]. Additionally, *Lactobacillus* relieves pancreatic β-cell dysfunction, which helps ameliorate insulin resistance [36,37] while decreasing the Bacteroides/Bacteroidetes ratio [37], which is positively correlated with body weight and LEP levels [38]. As a genus of butyric acid (a SCFAs)-producing bacteria, *Bacteroides* has a beneficial role in glucose metabolism and is negatively correlated with T2D [39]. Moreover, it can activate insulin resistance and dyslipidemia [40]. Additionally, *Bacteroides* are more abundant in non-obese individuals [40], suggesting that it may also have anti-obesity efficacy. The abundances of *Adlercreutzia* and *Allobaculum* are lower in mice on a high-fat diet [41,42]. As an active glucose assimilator, *Allobaculum* negatively correlates with liver and serum lipid levels [42], and can lessen systemic inflammation by reducing intestinal endotoxin in the blood [43]. Correlation analysis showed that the abundance of *Allobaculum* was negatively correlated with circulating LEP, while positively correlated with the expression of energy balance-related genes [44], indicating its potential contribution to improving insulin resistance. Some *Clostridium* spp. are regarded as probiotics [45], and genus *Clostridium* is positively correlated with INS sensitivity [46], suggesting its function in reducing insulin resistance. The relative abundance of *Anaerotruncus* increased after HFD treatment [42] and was positively correlated with both obesity-related indices and pro-inflammatory responses [47,48]. As a conditional opportunistic pathogen [47], *Anaerotruncus* shows a positive correlation with the levels of IL-6 [49].

On the other hand, HFD greatly influenced the plasma lipidome in mice, and 30 lipids were reversed by SPV treatment. Among them, many important lipids down-regulated by SPV belonged to SM, which is an essential source of Cer [50]. Cer has vital physiological functions. Various cellular stress inducers, such as inflammation, excessive saturated fatty acid intake, and chemical therapy, can increase the synthesis rate of Cer [51]. The accumulation of cellular Cers is associated with the development of obesity, diabetes, and various other illnesses [52]. Inhibitors of de novo Cer biosynthesis can reduce plasma Cer levels in obese mice and improve metabolism and inflammatory response [53]. In skeletal muscle, Cers can reduce AKT activity through two mechanisms: PKCζ and PP2A, resulting in insulin resistance [54].

As an upstream signaling component required for Cer biosynthesis induced by saturated fatty acids, TLR4 is closely related to Cer [55]. TLR4 is a component of the innate immune system and can recognize various molecular structures [56]. Recognition of saturated fatty acids by TLR4 triggers the activation of the innate immune signaling pathway, which is associated with increased inflammation in obesity and necessary for lipid-induced insulin resistance [55]. During the period of consuming a high-fat diet and being obese, the level of lipopolysaccharide (LPS) in the blood increases, leading to excessive activation of TLR4 and its binding with LPS [57]. This results in the production of numerous inflammatory mediators, triggering insulin resistance [58]. MyD88 is an adapter protein that binds to TLR4 through its Toll/IL-1 receptor domain, forming a complex and further activating other signaling molecules [59] such as TRAF6, which is closely related to the regulation of the INS signaling pathway [60]. Activation of TRAF6 can lead to the activation of IKK, resulting in phosphorylation and degradation of IκBα [61], releasing NF-κB and then translocating it into the nucleus [62]. NF-κB can regulate the expression of various inflammatory mediators (IL-1β, IL-6, TNF-α), inducing inflammation and immune response [63]. Moreover, SPV suppressed the levels of IL-18 and MCP-1 in the livers of HFD-induced mice. IL-18 activates the MyD88-NFκΒ signaling pathway by binding to its heterodimeric receptor (IL-18Rα/Rβ) [64]. The pro-inflammatory cytokine MCP-1, as a downstream signal of NF-κΒ, can be generated under the influence of IL-18 [65]. In obese patients, MCP-1 is involved in macrophage infiltration into adipose tissue as well as insulin resistance and hepatic steatosis [66]. Our results demonstrate that SPV regulates Cer levels to restore lipid metabolism. This regulatory effect subsequently inhibits the TLR4/NF-κB signaling pathway involved in obesity.

There are certain limitations in this study. We confirmed the anti-obesity and lipid-lowering effects of SPV; however, due to the complex composition of SPV, it remains unclear which specific components are responsible for these effects. Plasma lipidome analysis is limited in confirming changes in the mice; analyzing other molecules such as metabolites would provide clearer monitoring of the changes. Therefore, more studies need to be conducted.

## 5. Conclusions

In summary, SPV attenuated hepatic steatosis and inhibited the activity of inflammatory factors. Additionally, SPV protected mice from obesity by inhibiting Cer synthesis and suppressing signaling in the TLR4/NF-κB pathway, and this process may have some relevance to insulin resistance. These data offer experimental evidence supporting the use of SPV as a potential treatment for obesity.

## Data Availability

Data are contained within the article and Supplementary Materials.

## Supplementary Materials

The following supporting information can be downloaded at: https://www.mdpi.com/article/10.3390/nu16132159/s1, Figure S1: The mean areas of adipocytes of (A) iWAT, (B) eWAT, and (C) pWAT. The data are shown as the mean ± SD (*n* = 3). ^###^ *p* < 0.001 versus the vehicle-treated NCD-fed mice; ** *p* < 0.01, *** *p* < 0.001 versus the vehicle-treated HFD-fed mice; Figure S2: H&E staining of organs (heart, spleen, and kidney) in mice (200×; scale bar: 100 μm); Figure S3: (A) Alpha diversity analysis of gut microbiota. (B) Hierarchical clustering analysis of NCD, HFD, and HFD + SPV groups. (C) Principal Component Analysis (PCA) score plots of NCD, HFD, and HFD + SPV groups. ^#^ *p* < 0.05 versus the NCD group mice; Figure S4: Enlarged view of Figure 3D; Figure S5: Enlarged view of Figure 4A; Figure S6: Enlarged view of Figure 4B; Table S1: The information of ELISA kits used in biochemical detection; Table S2: The information of primary antibodies used in Western blotting; Table S3: The taxa with significance between the vehicle-treated HFD-fed mice and SPV-treated HFD-fed mice; Table S4: The differential metabolic pathways between the vehicle-treated HFD-fed mice and SPV-treated HFD-fed mice; Table S5: Basic information of 30 significantly regulated metabolites by SPV among experimental groups.
