# Supplementary material for: Anti-Obesity Activity of Sanghuangporus vaninii by Inhibiting Inflammation in Mice Fed a High-Fat Diet
Source: Nutrients. 2024 Jul 6;16(13):2159. doi: 10.3390/nu16132159 (PMC11243596; doi:10.3390/nu16132159)
Supplement: Supplementary file 1 [file nutrients-16-02159-s001.zip › nutrients-3066470-supplementary.pdf]

Supplementary data

Table S1. The information of ELISA kits used in biochemical detection.

Factors	Item numbers	Company
HDL-C	202923M	Ruixin Biological Technology Co., Ltd
LDL-C	202978M	Ruixin Biological Technology Co., Ltd
TC	CK-EN20870	Ruixin Biological Technology Co., Ltd
TG	CK-EN21757	Ruixin Biological Technology Co., Ltd
ALT	CK203034M	Ruixin Biological Technology Co., Ltd
AST	CK202596M	Ruixin Biological Technology Co., Ltd
TNF-α	CK20852M	Ruixin Biological Technology Co., Ltd
IL-1β	CK203063M	Ruixin Biological Technology Co., Ltd
IL-18	CK-E20324	Enzyme-linked Biotechnology Co., Ltd
IL-6	CK203049M	Ruixin Biological Technology Co., Ltd
MCP-1	ML037840	Enzyme-linked Biotechnology Co., Ltd
LEP	KT2619-A	Enzyme-linked Biotechnology Co., Ltd
Insulin	FY2579-A	Jiangsu Kete Biotechnology Co., Ltd
Ceramide	CK202647M	Ruixin Biological Technology Co., Ltd
FFA	MM-0326M1	Jiangsu Kete Biotechnology Co., Ltd

Table S2. The information of primary antibodies used in Western blotting.

Antibodies	Item numbers	Company	Dilution rate
TLR4	ab217274	Abcam	1/300
MyD88	bs-1047R	Bioss	1/2000
TRAF6	ab33915	Abcam	1/2000
P-IKKα+β	ab194528	Abcam	1/2000
T-IKKα+β	ab55341	Abcam	1/2000
P-IκBα	ab194528	Abcam	1/2000
T-IκBα	ab55341	Abcam	1/2000
P-NFκB	ab76302	Abcam	1/2000
T-NFκB	ab32536	Abcam	1/2000
PP2A	ab32104	Abcam	1/5000
P-PKC	ab75837	Abcam	1/5000
T-PKC	ab32376	Abcam	1/2000
NLRP3	ab263899	Abcam	1/1000
Caspase1	ab74279	Abcam	1/2000
IL-1β	bs-812R	Bioss	1/2000
GAPDH	ab181602	Abcam	1/500

Table S3. The taxa with significance between the vehicle-treated HFD-fed mice and SPV-treated HFD-fed mice.

Taxa	Abundance	LDA score	*p*-value
Down-regulated flora after SPV administration (Number: 7)			
Bacteria.Firmicutes	5.9887	5.3865	0.0273
Bacteria.Firmicutes.Clostridia	5.9378	5.3096	0.0273
Bacteria.Firmicutes.Clostridia.Clostridiales	5.9378	5.2988	0.0273
Bacteria.Firmicutes.Clostridia.Clostridiales.Ruminococcaceae	5.4697	4.9793	0.0390
Bacteria.Firmicutes.Clostridia.Clostridiales.Ruminococcaceae.Anaerotruncus	3.3972	3.2371	0.0265
Bacteria.Firmicutes.Erysipelotrichi.Erysipelotrichales.Erysipelotrichaceae.Clostridium	4.6852	4.3488	0.0273
Bacteria.Firmicutes.Erysipelotrichi.Erysipelotrichales.Erysipelotrichaceae.Clostridium.Clostridium_cocleatum	4.6784	4.3709	0.0273
Up-regulated flora after SPV administration (Number: 16)			
Bacteria.Actinobacteria	5.0180	4.6221	0.0273
Bacteria.Firmicutes.Bacilli	5.3203	4.9538	0.0273
Bacteria.Firmicutes.Bacilli.Lactobacillales.Lactobacillaceae	4.8476	4.3622	0.0273
Bacteria.Firmicutes.Bacilli.Lactobacillales.Lactobacillaceae.Lactobacillus	4.8420	4.3574	0.0273
Bacteria.Firmicutes.Clostridia.Clostridiales.Eubacteriaceae	2.4544	2.6013	0.0496
Bacteria.TM7	3.6113	3.2794	0.0265
Bacteria.TM7.TM7_3	3.6113	3.2707	0.0265
Bacteria.TM7.TM7_3.CW040	3.6113	3.2930	0.0241
Bacteria.TM7.TM7_3.CW040.F16	3.6113	3.2783	0.0241
Bacteria.Actinobacteria.Actinobacteria	3.8993	3.5748	0.0273
Bacteria.Actinobacteria.Actinobacteria.Bifidobacteriales	3.8993	3.5996	0.0273
Bacteria.Actinobacteria.Actinobacteria.Bifidobacteriales.Bifidobacteriaceae	3.8993	3.6161	0.0273
Bacteria.Firmicutes.Clostridia.Clostridiales.Clostridiaceae.Clostridium.Clostridium_celatum	2.8287	2.6062	0.0241
Bacteria.Firmicutes.Clostridia.Clostridiales.Lachnospiraceae.Roseburia	2.2921	2.7769	0.0349
Bacteria.Firmicutes.Clostridia.Clostridiales.Peptostreptococcaceae	3.9015	3.5926	0.0273
Bacteria.Proteobacteria	5.0090	4.7225	0.0273

The standard for differential taxa is LDA > 2 and $p < 0.05$.

Table S4. The differential metabolic pathways between the vehicle-treated HFD-fed mice and SPV-treated HFD-fed mice.

Pathway	Description	logFC	SE	*p*-values	Adj *p*-values
Down-regulated pathways after SPV administration (Number: 6)					
PWY0-1533	methylphosphonate degradation I	1.0380	0.3810	0.0065	0.0413
PWY-7198	pyrimidine deoxyribonucleotides de novo biosynthesis IV	1.4750	0.4283	0.0006	0.0116
PWY-7210	pyrimidine deoxyribonucleotides biosynthesis from CTP	1.3420	0.4247	0.0016	0.0176
PWY-7315	dTDP-N-acetylthomosamine biosynthesis	1.2740	0.4871	0.0089	0.0519
PWY-7456	mannan degradation	0.7628	0.3781	0.0437	0.1439
TRPSYN-PWY	L-tryptophan biosynthesis	0.7581	0.3761	0.0438	0.1439
Up-regulated pathways after SPV administration (Number: 25)					
FASYN-ELONG-PWY	fatty acid elongation—saturated	-1.0210	0.3792	0.0071	0.0438
P23-PWY	reductive TCA cycle I	-0.7919	0.3654	0.0302	0.1140
P42-PWY	incomplete reductive TCA cycle	-0.9458	0.3674	0.0100	0.0559
PWY0-162	superpathway of pyrimidine ribonucleotides de novo biosynthesis	-0.9976	0.3825	0.0091	0.0522
PWY-5345	superpathway of L-methionine biosynthesis (by sulfhydrylation)	-0.8927	0.3912	0.0225	0.1117
PWY-5837	1,4-dihydroxy-2-naphthoate biosynthesis I	-1.4410	0.3846	0.0002	0.0046
PWY-5838	superpathway of menaquinol-8 biosynthesis I	-1.1280	0.3861	0.0035	0.0285
PWY-5840	superpathway of menaquinol-7 biosynthesis	-1.2000	0.3840	0.0018	0.0176
PWY-5861	superpathway of demethylmenaquinol-8 biosynthesis	-1.1870	0.3875	0.0022	0.0210
PWY-5863	superpathway of phylloquinol biosynthesis	-1.4270	0.3845	0.0002	0.0047
PWY-5897	superpathway of menaquinol-11 biosynthesis	-1.2220	0.3840	0.0015	0.0170
PWY-5898	superpathway of menaquinol-12 biosynthesis	-1.2220	0.3840	0.0015	0.0170
PWY-5899	superpathway of menaquinol-13 biosynthesis	-1.2220	0.3840	0.0015	0.0170
PWY-6125	superpathway of guanosine nucleotides de novo biosynthesis II	-1.0210	0.3808	0.0073	0.0440
PWY-6383	mono-trans, poly-cis decaprenyl phosphate biosynthesis	-1.4430	0.4960	0.0036	0.0291
PWY-6545	pyrimidine deoxyribonucleotides de novo biosynthesis III	-0.9736	0.3816	0.0107	0.0589
PWY-6749	CMP-legionaminate biosynthesis I	-1.6240	0.4822	0.0008	0.0139

PWY-6876	isopropanol biosynthesis	-1.2110	0.3871	0.0018	0.0176
PWY-6969	TCA cycle V (2-oxoglutarate:ferredoxin oxidoreductase)	-0.8172	0.3713	0.0278	0.1140
PWY-7184	pyrimidine deoxyribonucleotides de novo biosynthesis I	-1.0820	0.3808	0.0045	0.0323
PWY-7196	superpathway of pyrimidine ribonucleosides salvage	-1.0870	0.3828	0.0045	0.0323
PWY-7197	pyrimidine deoxyribonucleotide phosphorylation	-1.1360	0.3827	0.0030	0.0251
PWY-7228	superpathway of guanosine nucleotides de novo biosynthesis I	-1.0460	0.3808	0.0060	0.0395
PWY-7377	cob(II)yrinate a,c-diamide biosynthesis I (early cobalt insertion)	-1.7460	0.3992	0.0000	0.0004
TCA	TCA cycle I (prokaryotic)	-1.0130	0.3756	0.0070	0.0438

LogFC: log2 (fold change); Adj *p*-values: *p*-values corrected by FDR.

Table S5. Basic information of 30 significantly regulated metabolites by SPV among experimental groups.

Lipid	Mean of vehicle-treated NCD mice	Mean of vehicle-treated HFD mice	Mean of SPV (1000 mg/kg)-treated HFD mice	vehicle-treated HFD mice versus vehicle-treated NCD mice		SPV (1000 mg/kg)-treated HFD mice versus vehicle-treated HFD mice	
				VIP	*p*-value	VIP	*p*-value
Up-regulated lipids after SPV administration (Number: 21)							
ChE(16:0)	25965957.1233	15109042.9926	18126795.8978	1.9027	0.0150	2.1045	0.0083
LPC(18:2)	18737705.9982	7397124.2198	8993413.2803	2.0475	0.0000	1.4905	0.0218
LPC(20:5)	18737705.9982	7397124.2198	8993413.2803	2.0475	0.0000	1.4905	0.0218
LPE(16:0)	7673903.6689	4142215.9153	5150348.5010	1.1384	0.0002	1.1930	0.0177
LPE(18:2)	9221615.4532	2253453.0076	3751704.7063	1.6018	0.0001	1.4596	0.0157
MePC(31:0e)	7302396.8759	2029600.6761	3676891.4196	1.3906	0.0002	1.5375	0.0134
MePC(32:4e)	12446399.6694	7125401.3521	8346184.2073	1.3933	0.0004	1.2999	0.0236
MePC(34:4e)	10088807.0510	6623831.5671	7884252.4925	1.1068	0.0034	1.3405	0.0150
MePC(35:4e)	15050128.9454	6024808.9368	7137862.4762	1.8248	0.0001	1.2469	0.0208
PC(15:0_18:2)	18057989.0580	2230595.4231	3621193.1207	2.4164	0.0001	1.4239	0.0097
PC(16:0_20:4)	12928434.9772	4761858.4129	6175952.0166	1.7366	0.0000	1.4575	0.0042
PC(18:1_18:1)	21896129.5830	11325018.1164	13349453.3146	1.9687	0.0002	1.7817	0.0002
PC(18:1_18:2)	13855252.8661	5447621.4618	7774903.2435	1.7358	0.0018	1.8431	0.0095
PC(34:2e)	18944096.8798	7933617.7833	10217459.0768	2.0124	0.0001	1.7559	0.0319
PC(34:3e)	7299017.9609	2017601.8140	3654010.2521	1.3921	0.0002	1.5340	0.0129
PC(38:7e)	15051157.8417	6041684.3065	7137891.0939	1.8230	0.0001	1.2317	0.0236
PC(40:5)	19728868.7159	14924992.0945	17310617.9023	1.2664	0.0147	1.8674	0.0091
PE(16:0e)	8162163.8447	4110414.8100	5125240.9137	1.1890	0.0053	1.1884	0.0219
PE(16:0p_22:6)	12363048.5450	7116224.9914	8540883.7865	1.3847	0.0004	1.4165	0.0184
PE(18:0p_22:6)	10088807.0510	6623831.5671	7884252.4925	1.1068	0.0034	1.3405	0.0150
PE(18:2e)	9221615.4532	2253453.0076	3751704.7063	1.6018	0.0001	1.4596	0.0157
Down-regulated lipids after SPV administration (Number: 9)							

LPC(20:3)	60080281.3532	74448074.7969	57459778.2716	2.1458	0.0268	4.8317	0.0258
MePC(33:1)	844783917.2133	1068451877.2706	997306135.0718	9.0885	0.0000	10.5065	0.0008
MePC(37:4)	3779345.6628	12397612.1449	10311818.0647	1.7849	0.0000	1.6540	0.0427
PC(36:4)	845335627.7563	1043607252.5341	991506130.3874	8.5066	0.0004	8.3913	0.0315
SM(d34:1)	183413602.5249	246656465.4006	188559592.0815	4.7490	0.0023	9.5444	0.0002
SM(d36:1)	13570393.6972	20511691.3014	12692734.0659	1.5707	0.0027	3.4813	0.0008
SM(d36:2)	6759562.7494	12828610.2660	9527872.1579	1.4850	0.0007	2.2474	0.0019
SM(d42:5)	987071.0230	28584187.0594	9518841.6589	3.1817	0.0002	5.4327	0.0009
SM(d42:6)	326727.6028	10965520.9760	6253413.4508	1.9734	0.0003	2.6428	0.0064

The standard for differential lipids is $p < 0.05$ and VIP > 1.0.

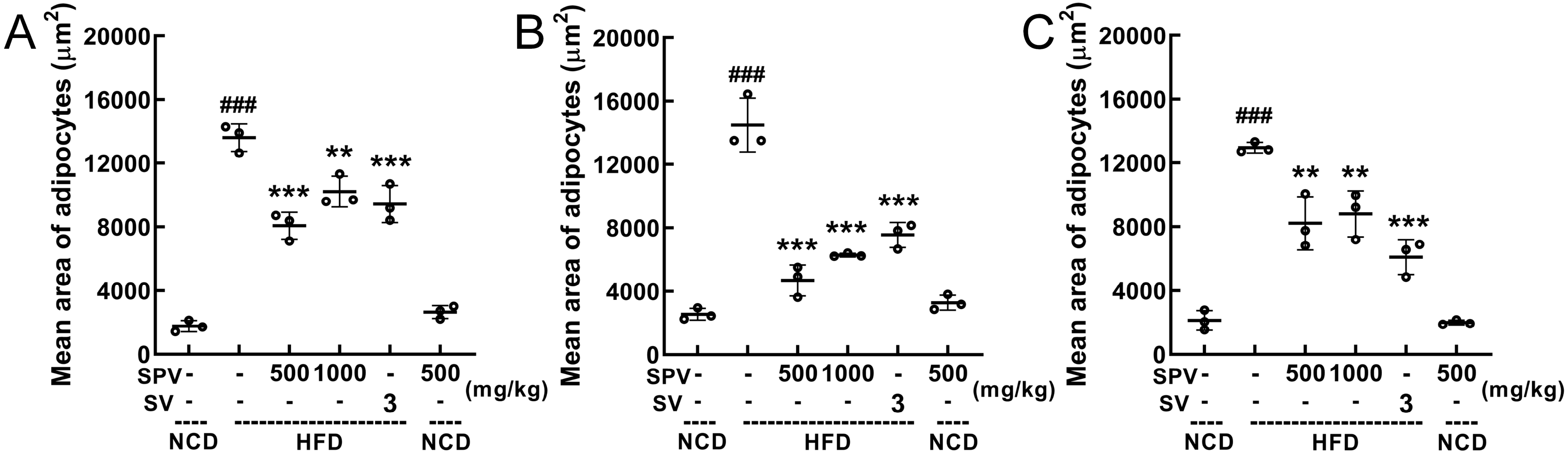

Figure S1. The mean areas of adipocytes of (A) iWAT, (B) eWAT, and (C) pWAT. The data are shown as the mean ± SD (n = 3). $^{###}p < 0.001$ versus the vehicle-treated NCD-fed mice; $^{**}p < 0.01$, $^{***}p < 0.001$ versus the vehicle-treated HFD-fed mice.

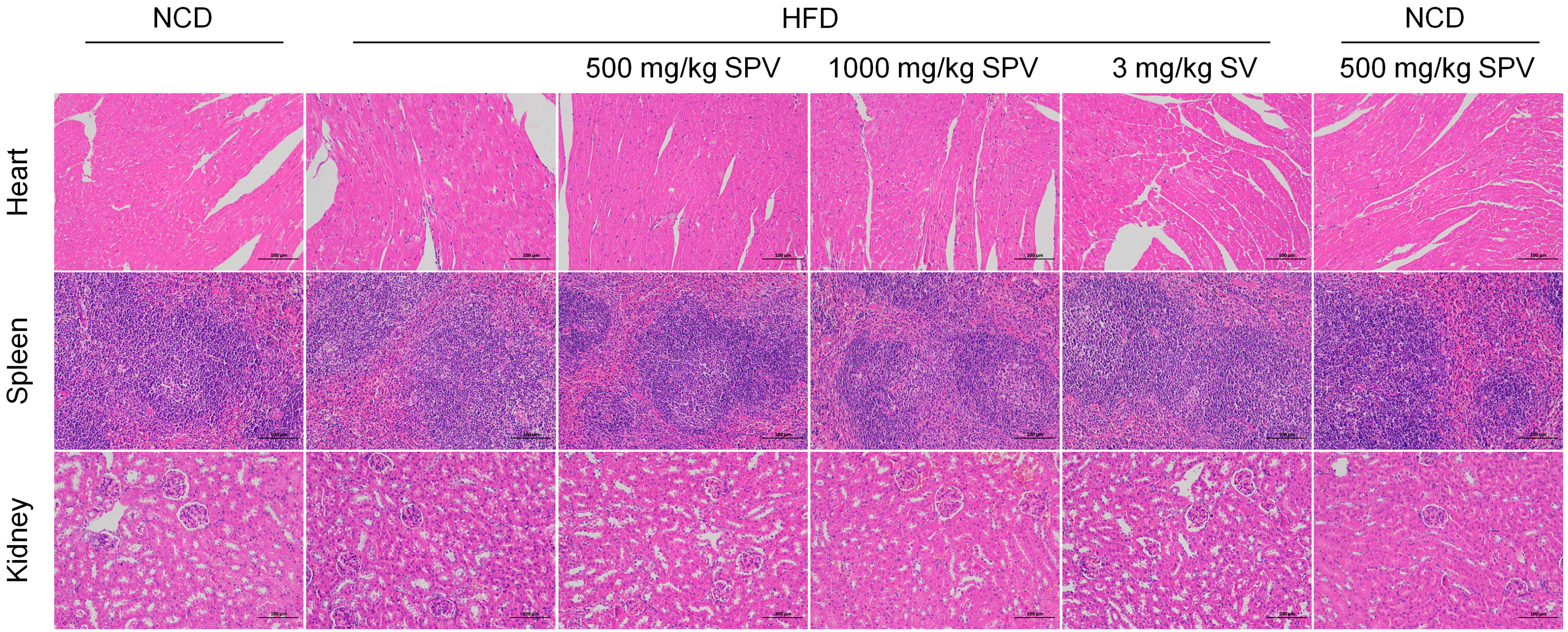

Figure S2. H&E staining of organs (heart, spleen, and kidney) in mice (200×; scale bar: 100 μm).

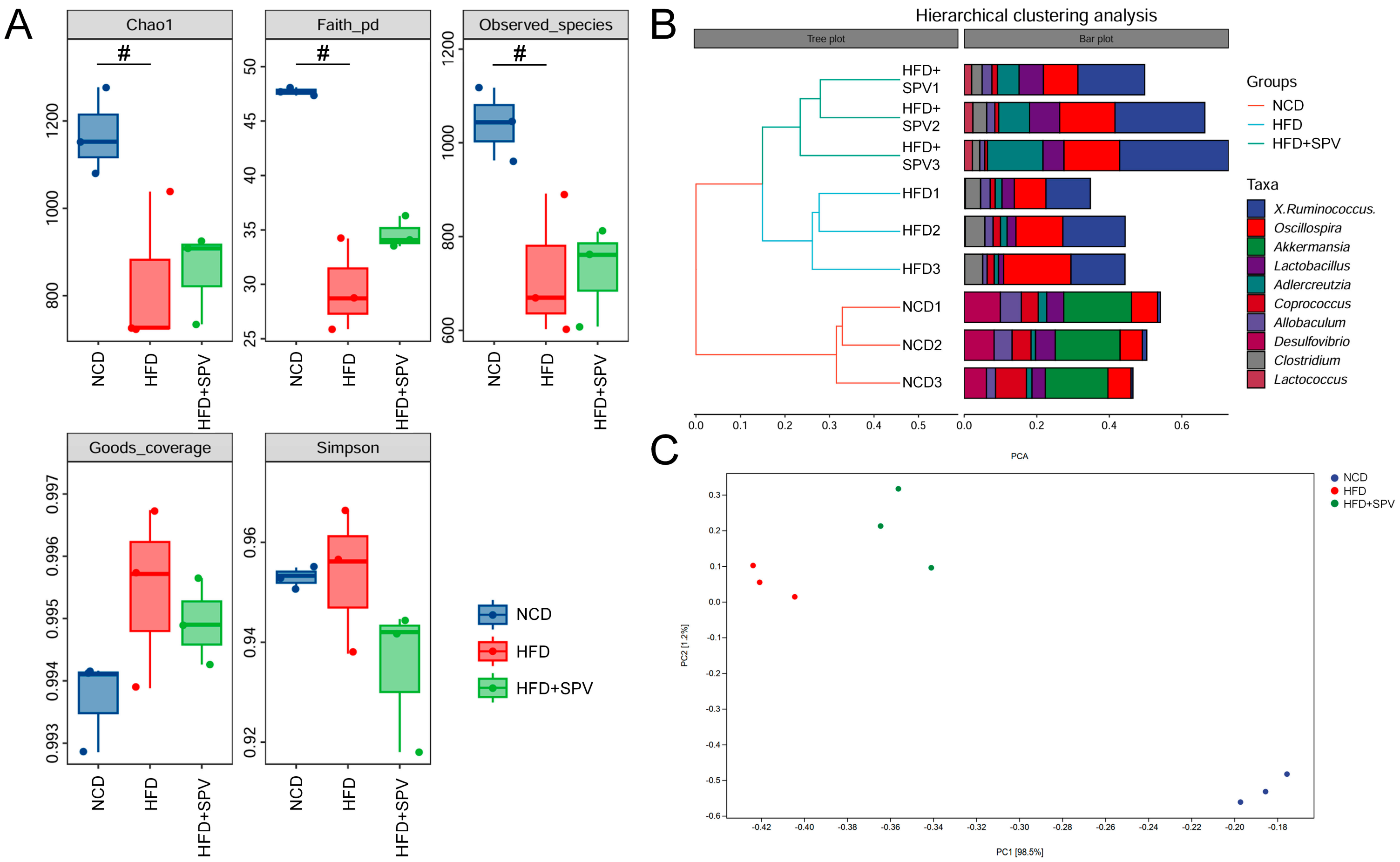

Figure S3. (A) Alpha diversity analysis of gut microbiota. (B) Hierarchical clustering analysis of NCD, HFD, and HFD+SPV groups. (C) Principal Component Analysis (PCA) score plots of NCD, HFD, and HFD+SPV groups. $^{\#}$ $p < 0.05$ versus the NCD group mice.

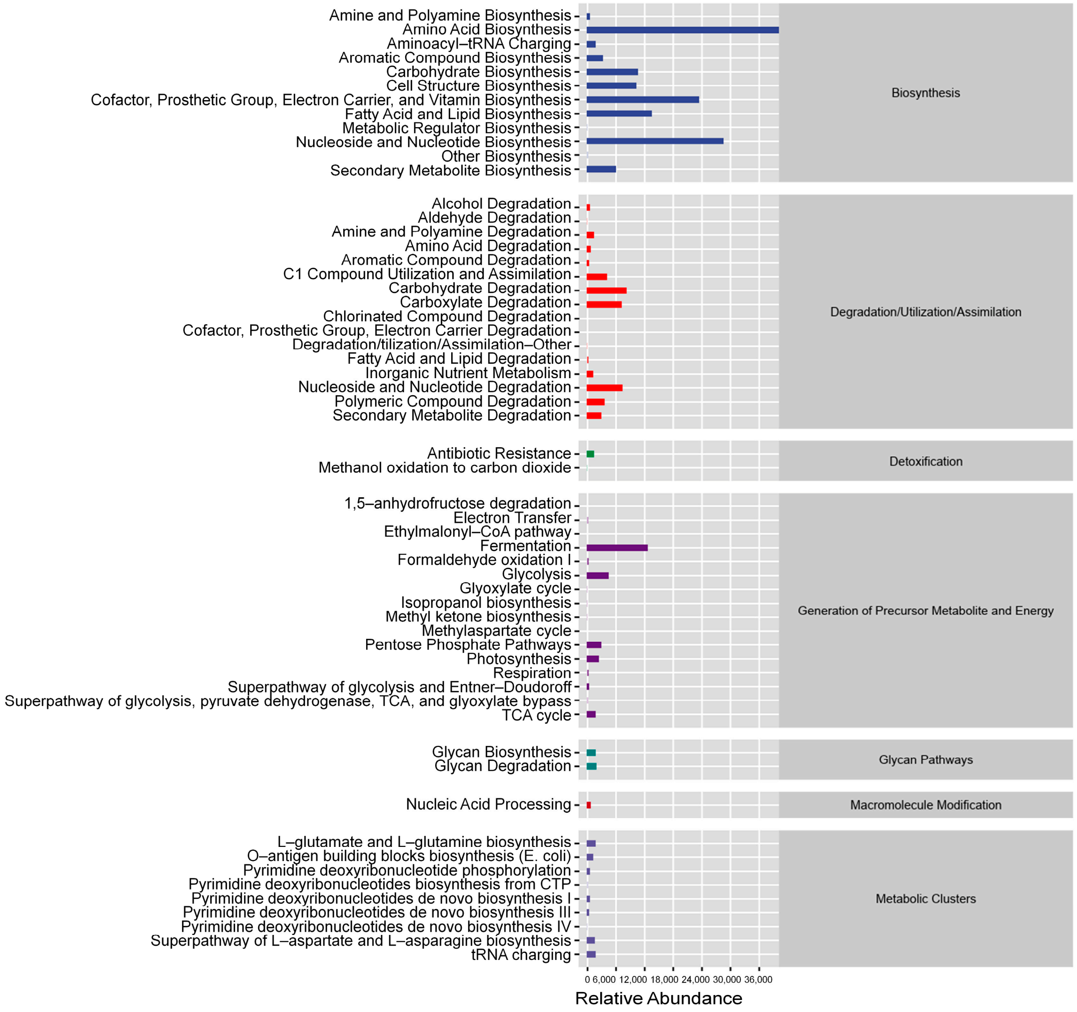

Figure S4. Enlarged view of Fig 3D.

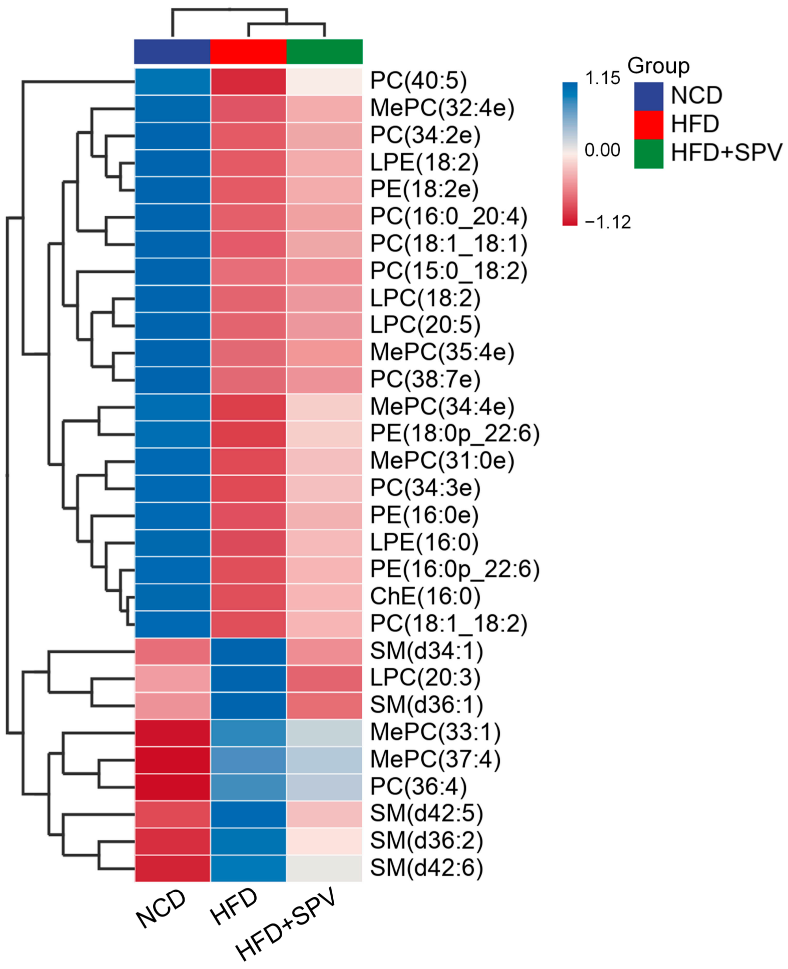

Figure S5. Enlarged view of Fig 4A.

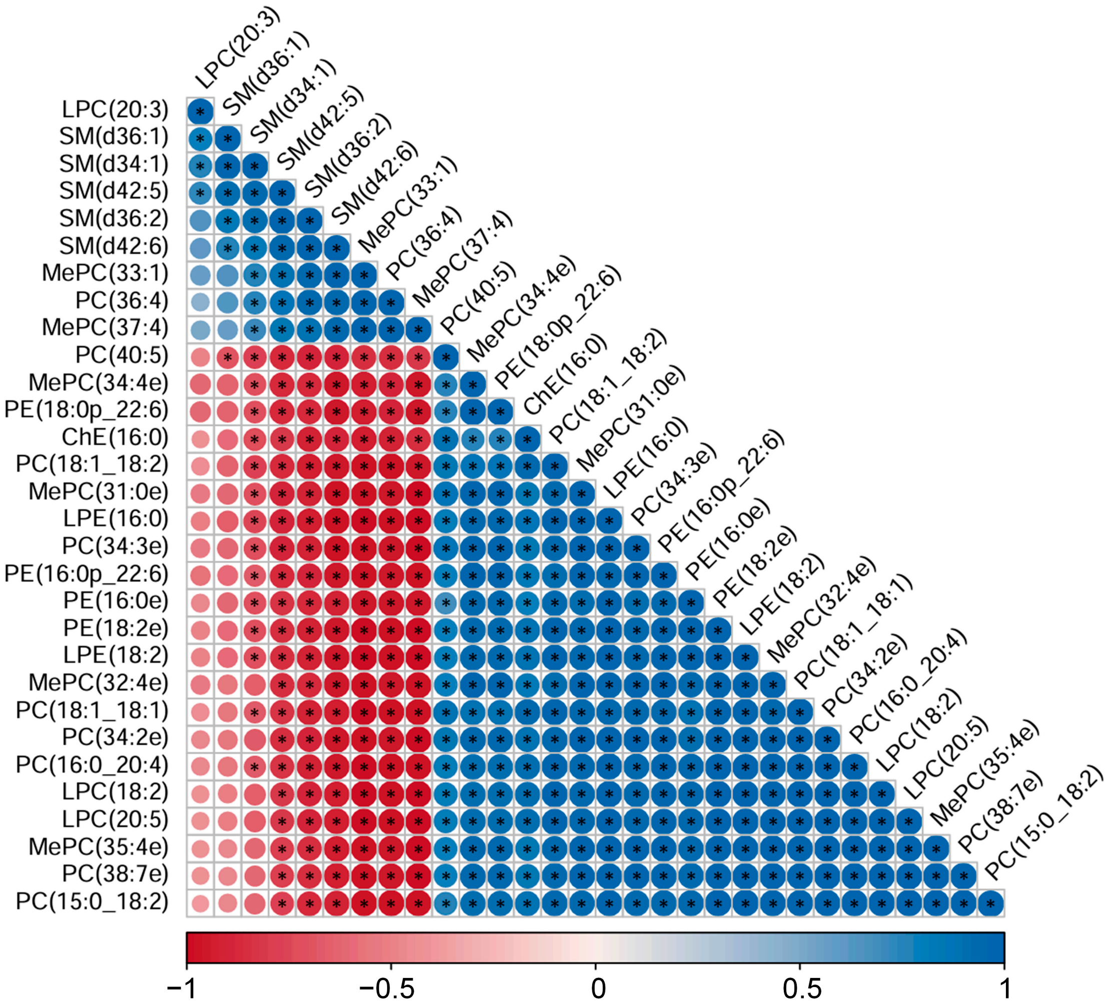

Figure S6. Enlarged view of Fig 4B.
